# Low-Cost Metamaterial Antennas: Forward-Looking Imaging Experiment and Analysis

**DOI:** 10.3390/s22155563

**Published:** 2022-07-26

**Authors:** Feng Ruan, Liang Han, Baoheng Zhang, Yachao Li, Liang Guo

**Affiliations:** 1 School of Physics and Optoelectronic Engineering, Xidian University, Xi’an 710071, China; 1605310269@stu.xidian.edu.cn (F.R.); 21051212273@stu.xidian.edu.cn (B.Z.); lguo@mail.xidian.edu.cn (L.G.); 2 National Laboratory of Radar Signal Processing, Xidian University, Xi’an 710071, China; ycli@mail.xidian.edu.cn

**Keywords:** correlated imaging, metamaterial antenna, error compensation, compressed sensing

## Abstract

A phase error correction method is proposed to compensate for the phase error in super-resolution correlated imaging based on metamaterial antennas. The varying carrier frequencies of a metamaterial antenna can generate the random radiation field for super-resolution correlation imaging, but the variation of the signal carrier frequency leads to large phase errors in the imaging results. In this proposed method, the sampling matrix in the super-resolution correlated imaging algorithm is used to compensate for the phase errors. Each element of the matrix is multiplied by a compensation phase corresponding to the phase error, and the error is subtly removed from the algorithm. In the experiment, the antenna pattern at each frequency of the metamaterial antenna is measured and recorded. In addition, an external field experiment is also carried out, and the collected data are imaged with the improved algorithm. Experimental results show that this technology can effectively solve the effect of phase errors on imaging results caused by signal carrier frequency changes.

## 1. Introduction

Synthetic aperture radar (SAR) obtains high-resolution images by the relative motion in the distance direction between the radar carrier and the target. However, since there is no relative motion in forward-looking imaging, SAR cannot be used to image the target. In this case, the forward-looking imaging of targets requires radar correlation imaging techniques. The most important step in a correlation imaging technique is the generation of the random radiation field. The random radiation field can be generated by a phased antenna array with modulated random phase shift. In the array, each antenna transmits the random phase signal to achieve high-resolution forward-looking imaging. However, it is complicated and costly to produce phased array antennas. Metamaterial antennas can obtain a fixed and constant random radiation field at a specified carrier frequency, so metamaterial antennas can replace phased array antennas to obtain high-quality forward-looking imaging results [1,2]. However, metamaterial antennas need to use signals with varying carrier frequencies, which causes a phase error in the signal echoes.

The super-resolution correlation imaging algorithm on compressed sensing can be used to process the echo signals of the phased array antenna to perform fast imaging with high resolution. However, when a phased array antenna is replaced by a metamaterial antenna, the algorithm cannot achieve imaging normally due to the phase error introduced into the echo signals [3]. For this reason, an improved super-resolution correlation imaging algorithm based on compressed sensing is proposed to eliminate the phase errors of the signal echoes received by metamaterial antennas, which can improve the imaging quality and efficiency.

## 2. Metamaterial Antennas

### 2.1. Metamaterial Antenna Aperture-Encoded Super-Resolution Imaging Mechanism

The study of microwave metamaterials begins with the verification of various special phenomena at various phases using a variety of different metamaterial units and then attempts to explain their properties using existing theories. In the area of metamaterial aperture imaging, the focus is on the tunable and broadband properties of metamaterial units [4].

The reconstruction of target images can be achieved rapidly with metamaterial antennas. The incoherent radiation pattern at each frequency of the metamaterial antenna can be used to quickly obtain incoherent detection data of the target through only a single detection. Then the target data are processed immediately by fast imaging algorithms to complete the reconstruction of the target image [5,6]. Therefore, the reconstruction of the target image can be completed in a short additional time.

**Figure 1:**
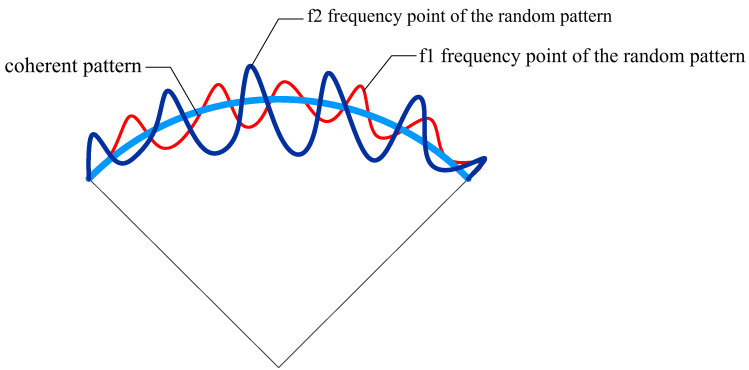
Comparison of coherent pattern of conventional antenna and random pattern of metamaterial antenna.

The metamaterial antenna in super-resolution imaging usually refers to a two-dimensional modulation array of metamaterials formed by a combination of metamaterial units. Compared with conventional antennas, a metamaterial modulation array can be flexibly designed to achieve electromagnetic wave spatial modulation [7,8,9,10,11]. Theoretical and experimental results show that metamaterials have good performance in refraction, polarization rotation, asymmetric transmission and broadband design [12,13]. The radiation gain distribution of the metamaterial antenna at each frequency can be designed randomly by using the broadband characteristics of the metamaterial unit with the feed structure design, that is, the random radiation pattern (as shown in Figure 1) [14,15].

Metamaterials possess some special electromagnetic properties when compared to conventional materials.

(1) Negative refraction
  When a beam of light enters conventional materials, the light is refracted; the refracted light and the incident light are on either side of the normal line. Unlike conventional materials, in metamaterials, both the refracted light and the incident light are on the same side, which means metamaterials have negative refraction.
(2) Inverse Doppler effect
  In conventional materials, when the transmitter and receiver start to become closer, the frequency of the electromagnetic wave received increases. However, in metamaterials, when the transmitter and receiver start to become closer, the frequency of the electromagnetic wave received increases.
(3) Inverse Cherenkov effect
  In conventional materials, the index of refraction is positive, so the electromagnetic waves radiated after the radiation is generated propagate forward. In metamaterials, however, the index of refraction is negative, so the electromagnetic waves radiated from radiation are propagated backwards.

### 2.2. Design of Broadband Metamaterial Randomly Coded Antenna

**Figure 2:**
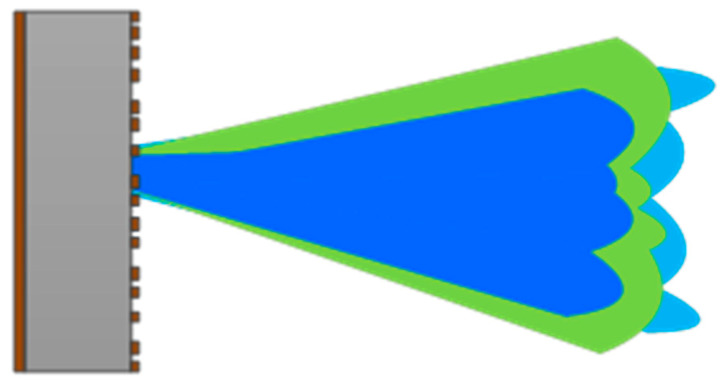
Radiation patterns of metamaterial antenna at different frequency points.

To ensure the performance of metamaterial antenna coded imaging, it is usually required that the broadband metamaterial antenna has certain directionality with randomness, statistically independent characteristics at each frequency within the broadband and consistency in the integrated direction [16,17,18,19]. Therefore, the design goal of the metamaterial antennas is to design random radiation patterns at discrete frequency points, as shown in Figure 2. There are two basic requirements for random radiation patterns:

(1). The patterns are random. The random radiation patterns at individual frequency points are not correlated within the desired range of detection angles;
(2). The patterns have certain directionality. Random radiation patterns have high gain within the bound angle and small gain or zero at the unwanted angle.

According to the design goals, three models of random phase distribution, random amplitude distribution and tunable hologram are usually used to carry out the randomly encoded metamaterial antenna design. The design of metamaterial antennas requires comprehensive consideration of performance index factors, such as antenna directionality, incoherent detection sample capacity and antenna integrated gain. Considering the need for antenna gain maximization, the spatial synthesis of electromagnetic waves using phase modulation has higher energy conversion efficiency and lower energy consumption than other models. Therefore, the design of random radiation metamaterial antenna based on random phase modulation is mainly discussed.

The random phase tuning broadband metamaterial antenna design method is summarized as follows: firstly, the broadband metamaterial unit is designed. Through the structure design of the metamaterial unit, the equivalent radiation phase of the metamaterial unit can traverse the design phase range in the broadband range. Secondly, the combination of metamaterial units with different equivalent radiation phases is randomly deployed in blocks to achieve random encoding of the metamaterial antenna aperture and complete the design of the broadband metamaterial random encoding antenna.

### 2.3. Implementation and Testing of Broadband Metamaterial Random-Coded Antenna

Since the metamaterial structure is etched on the microstrip lines, microstrip antennas with beam stacking effect are preferred. It is difficult to achieve the beam stacking effect with a single microstrip antenna, so an array of microstrip antennas is considered in the design [20,21,22,23,24].

**Figure 3:**
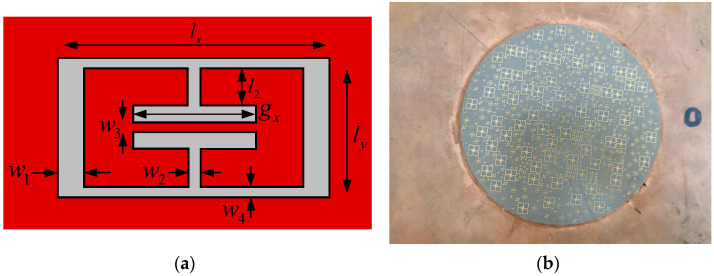
Metamaterial antenna model and physical object. (**a**) Metamaterial antenna model; (**b**) metamaterial antenna physical object.

Random radiation metamaterial antennas are realized by etching metamaterial structural units on the surface of a PCB board as a substrate. Metamaterial units with different resonance frequencies are randomly distributed on the surface of the PCB board. The energy of the guided wave structure is coupled by the different metamaterial resonant units and radiated to free space, resulting in a two-dimensional antenna aperture that can operate at several different resonant frequencies. Due to the mutual coupling effect existing between the units, the resonant characteristics at different frequencies become irregularly varied. According to the above idea and design model, a set of Ka-band metamaterial antennas were processed and implemented, as shown in Figure 3. For each unit cell of the proposed metamaterial structure, $w_{1}$ = 0.2 mm, $w_{2}$ = $w_{3}$ = $w_{4}$ = 0.1 mm, $l_{x}$ = 2.2 mm, $l_{y}$ = 1.2 mm and $l_{2}$ = 0.35 mm. The position of each cell in the array was randomly distributed. Since the Ka-band antenna’s array was stationary, the azimuth of the Ka-band antenna’s array ranged from −90° to 90°. The radiation characteristics of the antenna’s array were tested.

**Figure 4:**
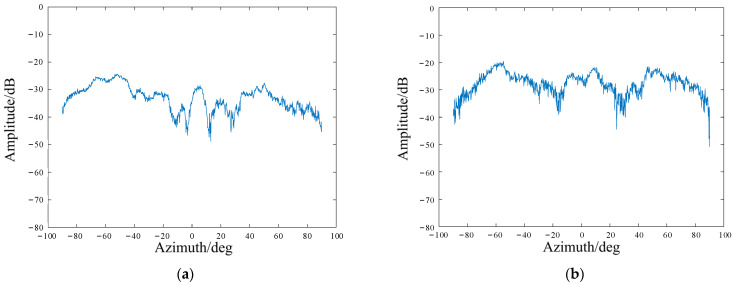

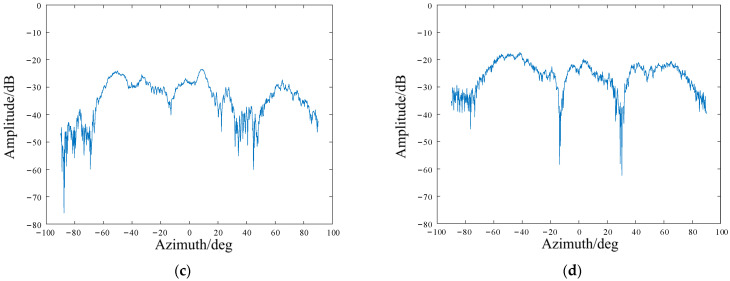
Random radiation field patterns at different frequencies. (**a**) 32.00 GHz; (**b**) 32.99 GHz; (**c**) 34.01 GHz; (**d**) 35.00 GHz.

A comparison of the measured radiation pattern of the metamaterial antenna at different frequencies is shown in the Figure 4. The radiation pattern characteristics of this transmissive metamaterial antenna were different at individual frequencies.

### 2.4. Improved Super-Resolution Correlated Imaging Algorithm

Since the carrier frequency of the signal used by a metamaterial antenna needs to be constantly changed, the echo signals received by different signal modes are different. The difference between the received echo signal of a single-frequency signal with constant carrier frequency and a single-frequency signal with carrier frequency stepping was analyzed.

The fundamental frequency echo of a single frequency signal with constant carrier frequency can be expressed as:

(1) $$\begin{array}{c} s_{s}\left(\hat{t},t_{q}\right)=\sum_{i\in V}\{\sigma_{i}F\left(\theta_{i},\beta_{i};t_{q}\right)rect\left(\frac{\hat{t}-2R_{i}\left(t_{q}\right)/c}{T_{p}}\right)\\ \exp\left(-j\frac{4\pi R_{i}\left(t_{q}\right)}{c}f_{c}\right)\} \end{array}$$

where $T_{p}$ is the pulse width, $t=\hat{t}+t_{q}$ is the full time, $\hat{t}$ is the intra-pulse fast time, $t_{q}=qT$ is the time when the $q_{th}$ pulse is transmitted and T is the pulse repetition period. $\theta_{i},\beta_{i}$ denote the azimuth and pitch angles of a scattering point i in the scene with reference to the radar line of sight direction, respectively. $\sigma_{i}$ is the target scattering coefficient. $F\left(\theta_{i},\beta_{i};t_{q}\right)$ is the equivalent transmit radiation pattern corresponding to the direction when the antenna irradiates the scattering point i in the $q_{th}$ pulse, $R_{i}\left(t_{q}\right)$ is the distance between the scattering point i and the radar at time $t_{q}$ and V is the target scene.

The fundamental frequency echo of a stepped single-carrier frequency signal can be expressed as:

(2) $$\begin{array}{c} s_{f}\left(\hat{t},t_{q}\right)=\sum_{i\in V}\{\sigma_{i}F\left(\theta_{i},\beta_{i};t_{q}\right)rect\left(\frac{\hat{t}-2R_{i}\left(t_{q}\right)/c}{T_{p}}\right)\\ \exp\left(-j\frac{4\pi R_{i}\left(t_{q}\right)}{c}f_{c}\right)\exp\left(-j\frac{4\pi R_{i}\left(t_{q}\right)}{c}q\Delta f\right)\} \end{array}$$

where Δf is the frequency difference between the two adjacent transmitted carrier frequency signals.

**Figure 5:**
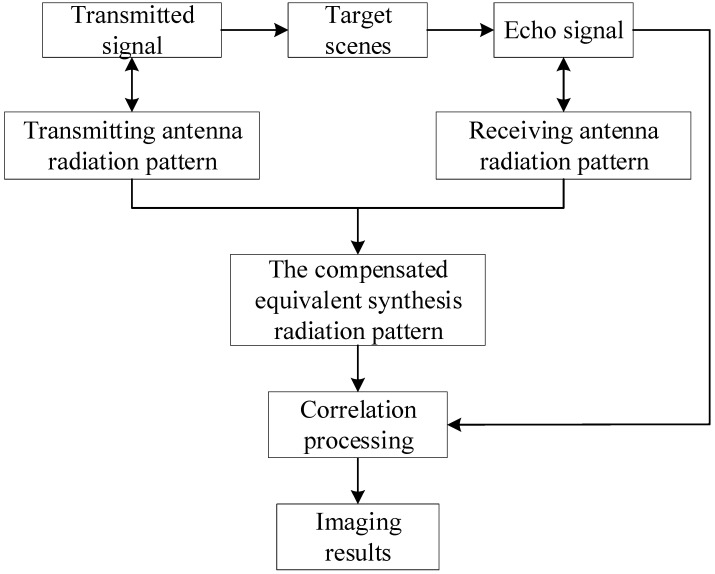
Flowchart of the improved correlation imaging algorithm.

It is obvious that Equation (2) has an extra $\exp\left(-j\left(4\pi R_{i}\left(t_{q}\right)/c\right)q\Delta f\right)$ in the phase when compared to Equation (1), and this item cannot simply be compensated by directly multiplying $\exp\left(j\left(4\pi R_{s}\left(t_{q}\right)/c\right)q\Delta f\right)$ ($R_{s}\left(t_{q}\right)$ is the distance between the center point of the scene and the radar at time $t_{q}$). A uniform compensation for this term results in a residual phase $\exp\left(j\left(4\pi /c\right)\left(R_{s}\left(t_{q}\right)-R_{i}\left(t_{q}\right)\right)q\Delta f\right)$ for each term in the echo. The Δf is large, although the $R_{s}\left(t_{q}\right)-R_{i}\left(t_{q}\right)$ item is close to zero. In addition, the Δf increases sharply when the number of pulses q is raised; thus, this residual phase cannot be ignored. Therefore, the traditional super-resolution correlation imaging algorithms based on compressed sensing cannot handle the echoes of the stepped single-frequency signal. We propose an improved super-resolution correlation imaging algorithm based on compressed sensing to handle the echoes of the stepped single-frequency signal. The flow of the improved algorithm is shown in Figure 5.

Figure 5 illustrates the flow of the improved super-resolution correlation imaging algorithm; the way to compensate the error is to discretely compensate the equivalent synthesis radiation pattern. The detailed flow of the algorithm is as follows:

In the work of radar, the transmitting field needs to be modulated by the transmitting antenna, the modulated transmitting field interacts with the target scattering field and the radar antenna receives the echoes after the interaction. The scene is discretized into multiple rectangular imaging grids. Assuming that the azimuthal equal-angle interval is Δθ and the pitch equal-angle interval is Δβ, the scene is divided into $N_{\theta }$ rectangular grid units in the azimuthal direction and $N_{\beta }$ rectangular grid units in the pitch direction, with the total number of rectangular grid units being $P=N_{\theta }\times N_{\beta }$. There is a point in the scene when its azimuth and elevation angles relative to the radar reference center are $\theta_{S}$ and $\beta_{S}$ and then the equivalent antenna radiation pattern can be expressed as $F\left(\theta_{S},\beta_{S},t\right)$, and when the backscattering coefficient can be expressed as $\sigma \left(\theta_{S},\beta_{S}\right)$, then the signal received by the radar can be expressed as:

(3) $$E_{r}\left(t\right)=\int_{S}\sigma \left(\theta_{S},\beta_{S}\right)F\left(\theta_{S},\beta_{S},t\right)d\theta_{S}d\beta_{S}$$

where S denotes the whole target scene, and the whole target scene is discretized to obtain:

(4) $${\left[\begin{array}{c} E_{r}\left(t_{1}\right)\\ E_{r}\left(t_{2}\right)\\ \vdots \\ E_{r}\left(t_{Q}\right) \end{array}\right]}_{Q\times 1}=F{\left[\begin{array}{c} \sigma_{1}\\ \sigma_{2}\\ \vdots \\ \sigma_{P} \end{array}\right]}_{P\times 1}$$

It can be simply expressed as:

(5) $$E_{r}=F\sigma$$

where Q is the number of samples, and $t_{q}$ is the $q_{th}$ discrete sampling moment (q=1,2,…,Q). $E_{r}$ is the vector composed of the sampled signals at each discrete moment, σ is the vector composed of the equivalent backward scattering coefficients of each unit after discretizing the scene and F is the sampling matrix after compensation. The matrix F is a Q×P matrix with the elements as shown in Equation (6).

(6) $$\left[\begin{array}{cccc} F\left(\theta_{1},\beta_{1};t_{1}\right)\exp\left(-j\frac{4\pi }{c}\cdot \Delta f\cdot R_{1}\left(t_{1}\right)\right) & F\left(\theta_{2},\beta_{2};t_{1}\right)\exp\left(-j\frac{4\pi }{c}\cdot \Delta f\cdot R_{2}\left(t_{1}\right)\right) & \cdots & F\left(\theta_{P},\beta_{P};t_{1}\right)\exp\left(-j\frac{4\pi }{c}\cdot \Delta f\cdot R_{P}\left(t_{1}\right)\right)\\ F\left(\theta_{1},\beta_{1};t_{2}\right)\exp\left(-j\frac{4\pi }{c}\cdot 2\Delta f\cdot R_{1}\left(t_{2}\right)\right) & F\left(\theta_{2},\beta_{2};t_{2}\right)\exp\left(-j\frac{4\pi }{c}\cdot 2\Delta f\cdot R_{2}\left(t_{2}\right)\right) & \cdots & F\left(\theta_{P},\beta_{P};t_{2}\right)\exp\left(-j\frac{4\pi }{c}\cdot 2\Delta f\cdot R_{P}\left(t_{2}\right)\right)\\ \vdots & \vdots & \ddots & \vdots \\ F\left(\theta_{1},\beta_{1};t_{Q}\right)\exp\left(-j\frac{4\pi }{c}\cdot Q\Delta f\cdot R_{1}\left(t_{Q}\right)\right) & F\left(\theta_{2},\beta_{2};t_{Q}\right)\exp\left(-j\frac{4\pi }{c}\cdot Q\Delta f\cdot R_{2}\left(t_{Q}\right)\right) & \cdots & F\left(\theta_{P},\beta_{P};t_{Q}\right)\exp\left(-j\frac{4\pi }{c}\cdot Q\Delta f\cdot R_{P}\left(t_{Q}\right)\right) \end{array}\right]$$

The phase $\exp\left(-j4\pi /c\cdot q\Delta f\cdot R_{p}\left(t_{q}\right)\right)$ is added to the discretized scene in the sampling matrix F in a one-to-one correspondence to compensate for the residual phase. $R_{p}\left(t_{q}\right)$ is the distance of the $p_{th}$ discrete unit from the radar. In the case of single-frequency signals with constant frequency, $E_{r}$ in Equation (5) does not have the error phase, so the correct imaging results can be solved. If the signal carrier frequency changes, there is an extra error phase in the echo on the left side of the equation while there is no change on the right side of the equation. The scattering coefficient of the scene and the equivalent synthesis antenna pattern remain the same when the carrier frequency signal changes. This means that Equation (6) fails in solving the backward scattering coefficients. The idea of compensation is to compensate for the error phase by discretizing the equivalent synthesis antenna pattern on the right side of the equation so that the equation can still maintain the balance.

To satisfy the non-correlation of the radiation field in time, the number of samples Q must be smaller than the total number of the grid units P; thus, the matrix F is a non-full rank matrix. Then, in order to solve Equation (5), a sparse constraint is used to transform it into a nonlinear optimization problem as follows:

(7) $$\hat{\sigma }=\arg\underset{\sigma }{\min}\left\{{\left\|E_{r}-F\sigma \right\|}_{2}^{2}+\mu {\left\|\sigma \right\|}_{1}\right\}$$

If the unit size of the matrix imaging grid is smaller than the Rayleigh aperture resolution limit, the above equation can be solved to obtain the super-resolution imaging results.

## 3. Results

The radar parameters of the field experiment are shown in Table 1.

The experiments were carried out in eleven different scenes. Three different sizes of corner reflector were used in the experiment, which were large, medium and small. It should be noted that in order to enable the center of the scene to be displayed in the center of the figure, the distance 17 m from the radar was moved to the “0” scale during data processing. In other words, the “0” scale position of the ordinate indicates the distance 17 m from the radar. The negative direction means closer to the radar relative to this position, and the positive direction means further away from the radar relative to this position. Similarly, the abscissa indicates the azimuth, the position of the “0” scale indicates the center position of the radar line of sight, the direction to the negative number indicates to the left relative to the position and the direction to the positive number indicates to the right relative to the position.

The detail of each experimental scene and the imaging results in the experimental scenes were as follows.

**Figure 6:**
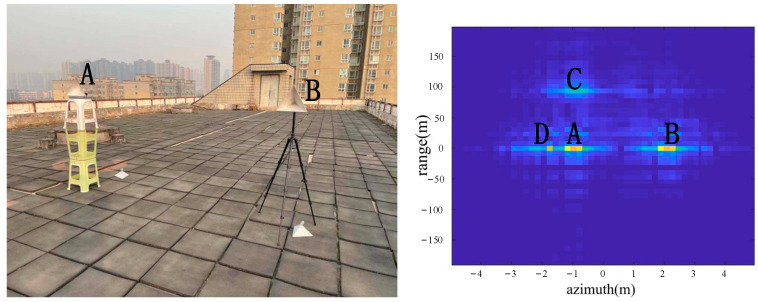
The 1st group of experimental scenes and imaging results.

As shown in Figure 6, the two corner reflectors A and B were placed 17 m from the radar. The two corner reflectors A and B were 2.5 m apart in the azimuth direction. The corner reflector A was on the left, and the corner reflector B was on the right. Both were big corner reflectors. The extra C in the above result picture was the position of the building about 110 m away from the radar. The color of C is not bright in the picture due to the long distance. D was an exhaust pipe on the left side of the platform about 20 m from the radar. According to the imaging results, the positions of A, B, C and D were consistent with the positions of objects in the actual scene.

**Figure 7:**
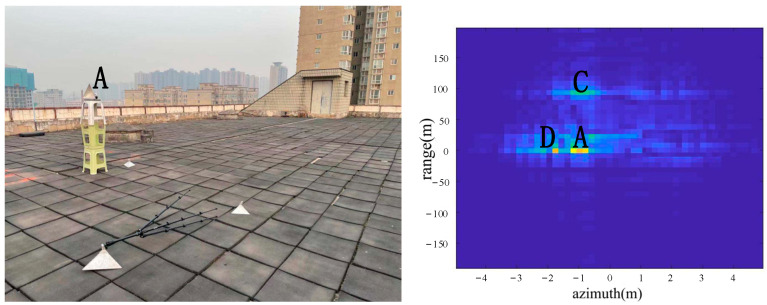
The 2nd group of experimental scenes and imaging results.

As shown in Figure 7, a corner reflector A was placed in the azimuth direction 17 m from the radar, and the corner reflector A was located to the left of the center position in the azimuth direction. A was a big corner reflector. Corner reflector B does not appear in the image because corner reflector B was lying on the ground. According to the imaging results, the positions of A, C and D were consistent with the positions of objects in the actual scene.

**Figure 8:**
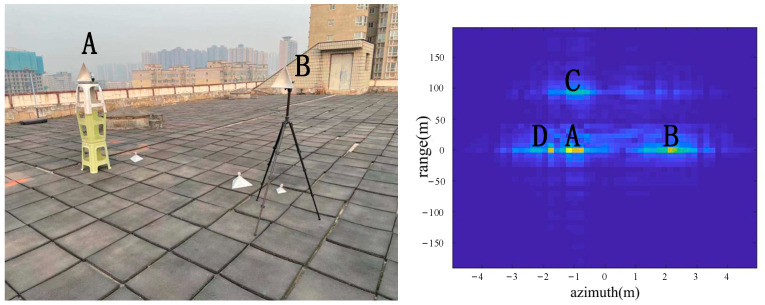
The 3rd group of experimental scenes and imaging results.

As shown in Figure 8, two corner reflectors A and B were placed in the azimuth direction 17 m from the radar. The two corner reflectors A and B were 2.5 m apart in the azimuth direction. The corner reflector A was on the left, and the corner reflector B was on the right. A was a big one, and B was a medium-sized one, so the reflection of A was stronger than the reflection of B. According to the imaging results, the positions of A, B, C and D were consistent with the positions of objects in the actual scene.

**Figure 9:**
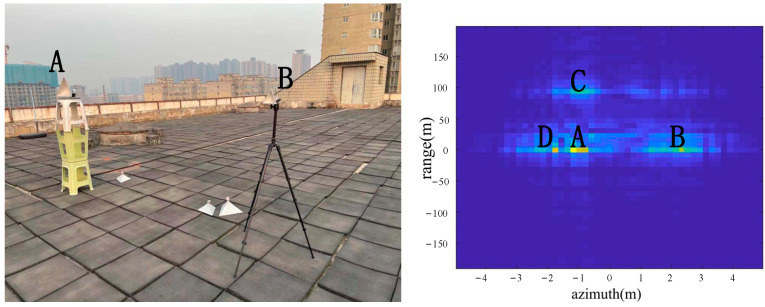
The 4th group of experimental scenes and imaging results.

As shown in Figure 9, two corner reflectors A and B were placed in the azimuth direction 17 m from the radar. The two corner reflectors A and B were 2.5 m apart in the azimuth direction. The corner reflector A was on the left, and the corner reflector B was on the right. A was a big one, and B was the small one, so the reflection of A was stronger than the reflection of B. According to the imaging results, the positions of A, B, C and D were consistent with the positions of objects in the actual scene.

**Figure 10:**
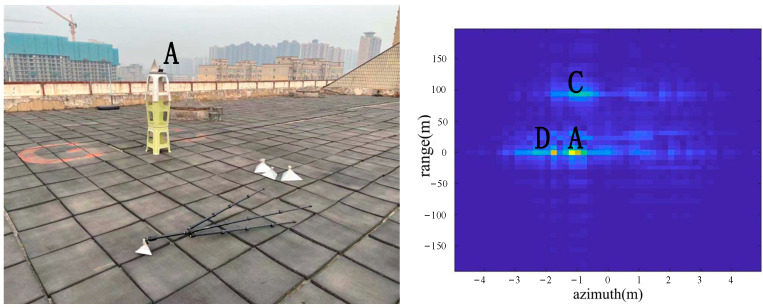
The 5th group of experimental scenes and imaging results.

As shown in Figure 10, a corner reflector A was placed in the azimuth direction 17 m from the radar, and the corner reflector A was positioned to the left of the center position in the azimuth direction. A was a medium-sized corner reflector, but the reflection of A was still obvious. Corner reflector B does not appear in the image because corner reflector B was lying on the ground. According to the imaging results, the positions of A, C and D were consistent with the positions of objects in the actual scene.

**Figure 11:**
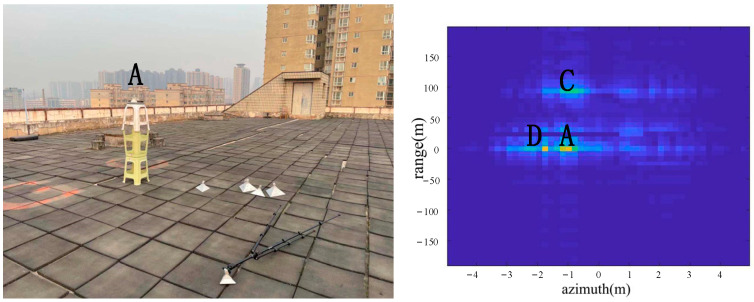
The 6th group of experimental scenes and imaging results.

As shown in Figure 11, a corner reflector A was placed in the azimuth direction 17 m from the radar, and the corner reflector A was placed to the left of the center of the azimuth direction. A was a small corner reflector; thus, the reflection of A was not very strong. According to the imaging results, the positions of A, C and D were consistent with the positions of objects in the actual scene.

**Figure 12:**
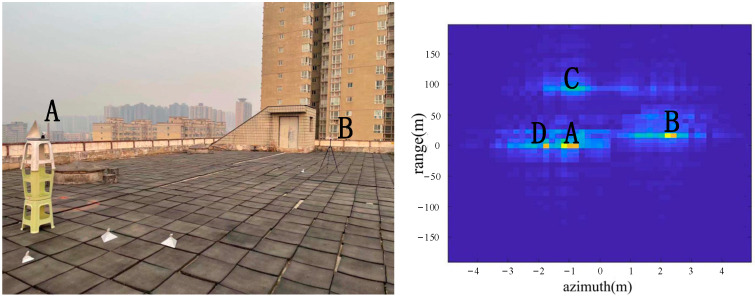
The 7th group of experimental scenes and imaging results.

As shown in Figure 12, two corner reflectors A and B were placed 17 m (left) and 25 m (right) from the radar, respectively. The two corner reflectors A and B were 2.5 m apart in the azimuth direction. A and B were both big corner reflectors so both A and B had strong reflections. In addition, the difference in distance between A and B was also reflected in the results. According to the imaging results, the positions of A, B, C and D were consistent with the positions of objects in the actual scene.

**Figure 13:**
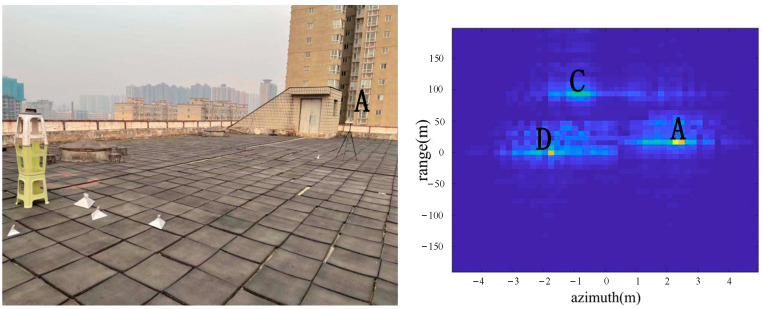
The 8th group of experimental scenes and imaging results.

As shown in Figure 13, a corner reflector A was placed in the azimuth direction 25 m from the radar, and the corner reflector A was placed to the right of the center position in the azimuth direction. A was a big corner reflector so the reflection of A was strong.

**Figure 14:**
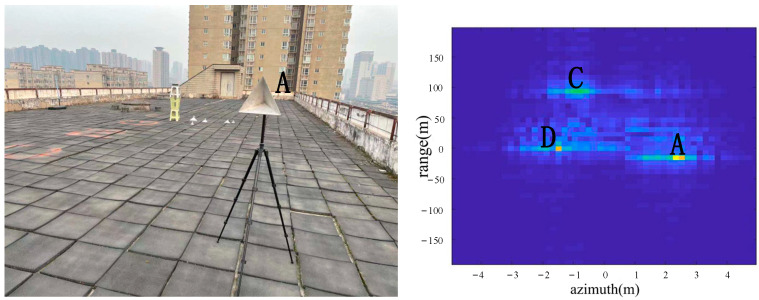
The 9th group of experimental scenes and imaging results.

As shown in Figure 14, the corner reflector A was placed in the azimuth direction 9 m from the radar, and the corner reflector A was placed to the right of the center position in the azimuth direction. A was a big corner reflector, so the reflection of A was strong. The ordinate of A was negative because the distance between the corner reflector A and the radar was less than 17 m.

**Figure 15:**
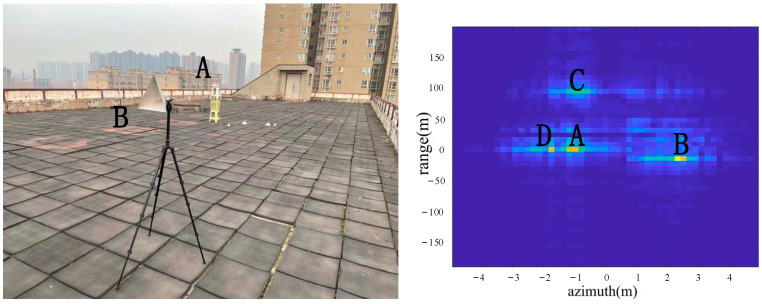
The 10th group of experimental scenes and imaging results.

As shown in Figure 15, two corner reflectors A and B were placed 17 m (left) and 9 m (right) from the radar, respectively. The two corner reflectors A and B were 2.5 m apart in the azimuth direction. The corner reflector A was on the left, 17 m in the distance, and the corner reflector B was on the right, 9 m in the distance. A and B were both big corner reflectors. According to the imaging results, the positions of A, B, C and D were consistent with the positions of objects in the actual scene.

**Figure 16:**
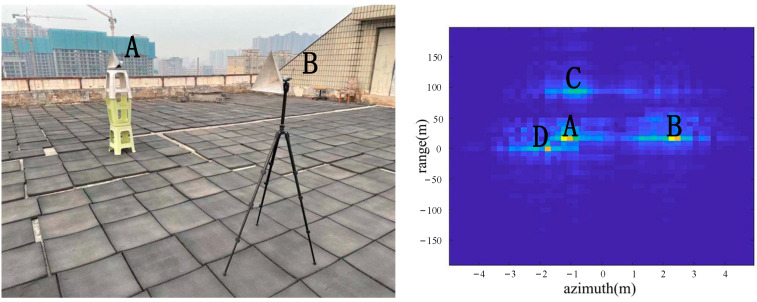
The 11th group of experimental scenes and imaging results.

As shown in Figure 16, two corner reflectors A and B were placed in the azimuth direction 25 m from the radar. The two corner reflectors A and B were 2.5 m apart in the azimuth direction. The corner reflector A was on the left, and the corner reflector B was on the right. A and B were both big corner reflectors. According to the imaging results, the positions of A, B, C and D were consistent with the positions of objects in the actual scene.

According to the simulation parameters in Table 1, the Rayleigh diffraction limit resolution was calculated to be 29.4449 m. In this experimental scenario, the distance between the two corner reflectors of A and B was 2.5 m. Therefore, the proposed algorithm achieved more than 10 times the super-resolution capability.

## 4. Discussion

From the comparison of Figure 6 and Figure 7, it can be seen that the algorithm has the basic ability to identify whether there is a corner reflector or not, and it is possible to correctly display the position of the angular reflection and the position of other objects with reference significance accurately. From the comparison of Figure 6, Figure 8 and Figure 9 or the comparison of Figure 7, Figure 10 and Figure 11, it can be seen that due to the different RCS of large, small and medium-sized corner reflectors, there was a certain difference in brightness and darkness in the results, but the difference was not very large. From the results of the 12th to 16th experiments, it can be seen that this algorithm also has a certain resolving power in distance. However, the signal bandwidth emitted by this radar device was not big enough due to some reasons. This resulted in poor radar resolution in the range. So only when two objects were far apart in the upward distance could they be distinguished. The effectiveness of the proposed algorithm was proved by the above field experiments using corner reflectors.

## 5. Conclusions

In general, the results of this experiment were relatively good. This experiment proved that the improved algorithm can not only realize super-resolution imaging but also has the ability to compensate phase errors. The result graphs of each group of experiments are relatively noisy in a comprehensive view, but the highlights are relatively clear.

## Figures and Tables

**Table 1:** Parameters of the radar used in the field experiment.

Experimental Parameters	Values
Radar working wavelength *λ*	0.0084~0.0094 m
$\mathrm{Initial}\;\mathrm{carrier}\;\mathrm{frequency}\;(\mathrm{center}\;\mathrm{frequency})\;f_{c}$	32 GHz
Frequency step ladder Δf	30 MHz
Antenna beam width *θ*	120 degrees
The distance between the center of the scene and the radar (slant distance) *R*	17 m
Pulse sampling times *Q*	128 times
$\mathrm{Pulse}\;\mathrm{width}\;T_{p}$	24 μs
Signal bandwidth *B*	15 MHz
$\mathrm{Sampling}\;\mathrm{frequency}\;F_{s}$	20 MHz
Carrier speed *v*	0 m/s
